# The sarcopenia and physical frailty in older people: multi-component treatment strategies (SPRINTT) project: description and feasibility of a nutrition intervention in community-dwelling older Europeans

**DOI:** 10.1007/s41999-020-00438-4

**Published:** 2021-02-13

**Authors:** S. K. Jyväkorpi, A. Ramel, T. E. Strandberg, K. Piotrowicz, E. Błaszczyk-Bębenek, A. Urtamo, H. M. Rempe, Ó. Geirsdóttir, T. Vágnerová, M. Billot, A. Larreur, G. Savera, G. Soriano, C. Picauron, S. Tagliaferri, C. Sanchez-Puelles, V. Sánchez Cadenas, A. Perl, L. Tirrel, H. Öhman, C. Weling-Scheepers, S. Ambrosi, A. Costantini, K. Pavelková, M. Klimkova, E. Freiberger, P. V. Jonsson, E. Marzetti, K. H. Pitkälä, F. Landi, R. Calvani, SPRINTT consortium, Roberto Bernabei, Claudio Boni, Vincenzo Brandi, Marianna Broccatelli, Riccardo Calvani, Carilia Celesti, Americo Cicchetti, Agnese Collamati, Silvia Coretti, Emanuela D’Angelo, Mariaelena D’Elia, Francesco Landi, Giovanni Landi, Maria Lorenzi, Luca Mariotti, Anna Maria Martone, Emanuele Marzetti, Elena Ortolani, Teodosio Pafundi, Anna Picca, Matteo Ruggeri, Sara Salini, Giulia Savera, Matteo Tosato, Davide L. Vetrano, Fabrizia Lattanzio, Renato Baldoni, Serena Bernabei, Anna Rita Bonfigli, Silvia Bustacchini, Barbara Carrieri, Laura Cassetta, Antonio Cherubini, Michela Cucchi, Giacomo Cucchieri, Anna Rita Costantini, Giuseppina Dell’Aquila, Emma Espinosa, Massimiliano Fedecostante, Riccardo Fraternali, Roberta Galeazzi, Antonella Mengarelli, Stefano Piomboni, Elena Posacki, Eddy Severini, Tiziana Tregambe, Fabiana Trotta, Marcello Maggio, Fulvio Lauretani, Valeria Buttò, Alberto Fisichella, Chiara Guareschi, Yari Longobucco, Sara Tagliaferri, Mauro Di Bari, Leocadio Rodriguez-Manas, Soledad Alamo, Cristina Alonso Bouzon, Jimmy Gonzales Turin, Olga LaosaLaosa Zafra, Andrea Lopez Picazo, Laura Pedraza Sepulveda, Juan Luis SanchezSanchez, Carlos Sanchez Puelles, Myryam Valdés Aragonés, Alfonso José CruzJentoft, Juan Alvarez Santos, Loreto Alvarez-Nebreda, Nelén Fernández JiménezJiménez, Jesús Mateos-del Nozal, Beatriz Montero-Errasquín, Beatriz PonceBeatriz Ponce Moreno, Cristina Roldán-Plaza, Alfonso Romera-de Vicente, Vicente Sánchez-Cadenas, Carmen Sánchez-Castellano, Elisabet Sánchez-García, Mará Nieves Vaquero-Pinto, Eva Topinková, Lucie Bautzka, Kamila Blechová, Terza Gueye, Ilona Juklicková, Terza Klbíková, JanaJana Křenková, Pavla Mádlová, Helena Mejstriková, Renata Melčova, Helena Michálková, Ingrid Ryznarová, Ivana Drastichova, Eva Hasaliková, Radim Hucko, Seget Jakub, Monika Janácová, Michaela Kilmková, Martina Parízková, Kristyna Pavelková, Michaela Redrova, PetraPetra Rusková, Cornel C. Sieber, Tina Auerswald, Christof Engel, Anna Franke, Ellen Freibergen, Ulrike Freiheit, Susann Gotthardt, Karin Kampe, Robert Kob, Christine Kokott, Carolin Kraska, Christian Meyer, Veronika Reith, Hanna Rempe, Daniel Schoene, Gabrielle Sieber, Kerstin Zielinski, Stefan D. Anker, Nicole Ebner, Romanus Grütz, Stephan von Haehling, Annemie M. W. J. Schols, Harry Gosker, Stephanie Huysmans, Sandy Quaaden, Jos M. Schols, Nick Smeets, Pascal Stevens, Coby van de Bool, Claire Weling, Timo Strandberg, Satu Jyväkorpi, Katja Hallikas, Marjatta Herranen, Tiina Huusko, Laura Hytönen, Kirsi Ikonen, Anne Karppi-Sjöblom, Kaisa Karvinen, Maija Käyhty, Tarja Kindsted, Erja Landström, Saana Leirimaa, Hanna Öhman, Kaisu Pitkälä, Anja Punkka, Anne-Mari Saavalainen, Tuulia Salo, Madis Sepa, Katja Sohlberg, Anele Urtamo, Emmi Vaatamoinen, Sirpa Venäläinen, Hannu Vanhanen, Bruno Vellas, Gabor Abellan Van Kan, Virginie Biville, Lauréane Brigitte, Carole Cervera, Matteo Cesari, Marie Champarnaud, Céline Cluzan, Muriel Croizet, Sophie Dardenne, Marie Dorard, Charlotte Dupuy, Emilie Durand, Catherine Faisant, Bertrand Fougère, Philippe Girard, Sophie Guyonnet, Emiel Hoogendijk, Rémi Mauroux, Agathe Milhet, Sylvie Montel, Pierre-Jean Ousset, Cécile Picauron, Gaelle Soriano, Maturin Tabue Teguo, Bernard Teysseyre, Sandrine Andrieu, Alessandro Blasimme, Cedric Dray, Emmanuelle Rial-Sebbag, Philippe Valet, Thierry Dantoine, Maxime Billot, Noelle Cardinaud, Muriel Castelli, Marion Charenton-Blavignac, Cecilia Ciccolari-Micaldi, Caroline Gayot, Anael Larreur, Cécilie Laubarie-Mouriet, Delphine Marchesseau, Thomas Mergans, Thai Binh Nguyen, Arnaud Papon, Johann Ribet, Isabelle Saulinier, Achille Tchalla, Thomas RappRapp, Nicolas Sirven, Anna Skalska, Ewa Blaszcyk, Marcin Cwynar, Joanna Czesak, Paulina Fatyga, Malgorzata Fedyk-Lukasik, Tomasz Grodzicki, Paulina Jamrozik, Zbigniew Janusz, Ewa Klimek, Sylwia Komoniewska, Maria Kret, Maciej Ozog, Agnieszka Parnicka, Kararzyna Petitjean, Anna Pietrzyk, Karolina Piotrowicz, Barbara Skalska-Dulinska, Damian Starzyk, Katarzyna Szczerbinska, Borys Witkiewicz, Anna Wlodarczyk, Alan Sinclair, Sital Harris, Allison Ogborne, Sarah Ritchie, Caroline Sinclair, Harriet Sinclair, Srikanth Bellary, Sital Harris, Hannah Worthington, Jaroslaw Derejczyk, Regina Roller-Wirnsberger, Pálmi Jónsson, Philippe Bordes, Sandrine Arnaud, Christian Asbrand, Raphael Bejuit, Sandrine Durand, Klaus Flechsenhar, Florence Joly, Regis Le Lain, Mathieu Moncharmont, Jerome Msihid, Aurèle Ndja, Brigitte Riche, Anne Caroline Weber, Jiazhao Yuan, Ronenn Roubenoff, Patrick Kortebein, Ram R. Miller, Carmen Gorostiaga, Patricia Belissa-Mathiot, Hao Hu, Laurence Laigle, Itziar Martinez Melchor, Alan Russel, Mike Bennecky, Tom Haws, Ashish Joshi, Karen Philpott, Anne Walker, Gianluca Zia, Sabina De Giorgi, Luca Feletti, Elisa Marchioro, Francesco Mocci, Maria Grazia Varesio, Alfredo Cesario, Barbara Cabin, Willem P. de Boer, Claire Ignaszewski, Ingrid Klingmann, Miriam Vollenbroek-Hutten, Termie Hermens, Stephanie Jansen-Kosterink, Monique Tabak, Patrick Blandin, Laure Coutard, Anne-Marie Lenzotti, Hocine Mokhtari, Nicolos Rodon

**Affiliations:** 1 Clinicum, Department of General Practice, Helsinki University Central Hospital, University of Helsinki, Tukholmankatu 8 B, 00014 Helsinki, Finland; 2 The Icelandic Gerontological Research Center, The National University Hospital of Iceland, Reykjavik, Iceland; 3 University of Oulu, Center for Life Course Health Research, Oulu, Finland; 4 Faculty of Medicine, Department of Internal Medicine and Gerontology, Jagiellonian University Medical College, Krakow, Poland; 5 Department of Nutrition and Drug Research, Institute of Public Health, Faculty of Health Science, Jagiellonian University Medical College, Krakow, Poland; 6 Institute for Biomedicine of Aging, Friedrich-Alexander-University of Erlangen-Nürnberg, Erlangen, Germany; 7 1St Faculty of Medicine, Department of Gerontology & Geriatrics, Charles University in Prague, General University Hospital Prague, Nové Město, Czech Republic; 8 PRISMATICS Lab (Predictive Research In Spine/Neuromodulation Management And Thoracic Innovation/Cardiac Surgery), Poitiers University Hospital, Poitiers, France; 9 Department of Geriatrics, University Hospital of Limoges, Limoges, France; 10 Università Cattolica del Sacro Cuore, Rome, Italy; 11 Gérontopôle, Centre Hospitalier Universitaire de Toulouse, Toulouse, France; 12 Department of Medicine and Surgery, University of Parma, Parma, Italy; 13 University Hospital Getafe, Madrid, Spain; 14 University Hospital Ramon Y Cajal Madrid, Madrid, Spain; 15 Medical University of Graz, Graz, Austria; 16 Diabetes Frail, Medici Medical Practice, Luton, UK; 17 Maastricht University Medical Center, Maastricht, The Netherlands; 18 IRCCS INRCA, Ancona, Italy; 19 Silesian Hospital, Opava, Czech Republic; 20 Fondazione Policlinico Universitario “A. Gemelli” IRCCS, Rome, Italy

**Keywords:** SPRINTT, Nutrition intervention, Protein intake, Energy intake, Nutrition counselling

## Abstract

**Background:**

The “Sarcopenia and Physical Frailty in Older People: Multicomponent Treatment Strategies” (SPRINTT) project sponsored a multi-center randomized controlled trial (RCT) with the objective to determine the effect of physical activity and nutrition intervention for prevention of mobility disability in community-dwelling frail older Europeans. We describe here the design and feasibility of the SPRINTT nutrition intervention, including techniques used by nutrition interventionists to identify those at risk of malnutrition and to carry out the nutrition intervention.

**Methods:**

SPRINTT RCT recruited older adults (≥ 70 years) from 11 European countries. Eligible participants (*n* = 1517) had functional limitations measured with Short Physical Performance Battery (SPPB score 3–9) and low muscle mass as determined by DXA scans, but were able to walk 400 m without assistance within 15 min. Participants were followed up for up to 3 years. The nutrition intervention was carried out mainly by individual nutrition counseling. Nutrition goals included achieving a daily protein intake of 1.0–1.2 g/kg body weight, energy intake of 25–30 kcal/kg of body weight/day, and serum vitamin D concentration ≥ 75 mmol/L. Survey on the method strategies and feasibility of the nutrition intervention was sent to all nutrition interventionists of the 16 SPRINTT study sites.

**Results:**

Nutrition interventionists from all study sites responded to the survey. All responders found that the SPRINTT nutrition intervention was feasible for the target population, and it was well received by the majority. The identification of participants at nutritional risk was accomplished by combining information from interviews, questionnaires, clinical and laboratory data. Although the nutrition intervention was mainly carried out using individual nutritional counselling, other assisting methods were used as appropriate.

**Conclusion:**

The SPRINTT nutrition intervention was feasible and able to adapt flexibly to varying needs of this heterogeneous population. The procedures adopted to identify older adults at risk of malnutrition and to design the appropriate intervention may serve as a model to deliver nutrition intervention for community-dwelling older people with mobility limitations.

**Supplementary Information:**

The online version contains supplementary material available at 10.1007/s41999-020-00438-4.

## Abstract (article highlights)

**Aim:**

To describe the methods and feasibility of the nutritional intervention carried out within the SPRINTT Randomized cotrolled trial. We also illustrate how nutrition interventionists identified participants at risk of malnutrition and the lessons learnt from the nutrition intervention.

**Findings:**

SPRINTT nutrition intervention was well-received by the majority of the participants. It was mainly carried out using tailored nutrition counselling, but also other means of delivering the intervention were successfully used. Compared with a standard nutrition prescription, an individualized protocol to diagnose malnutrition and follow-up by tailored nutrition counselling helped achieve nutritional targets more effectively in spite of diversity of population in nutritional habits and in some cases reluctance to accept changes.

**Message:**

The SPRINTT nutrition intervention was feasible and allowed flexibility to the varying needs and cultural differences of this heterogeneous population of frail, older Europeans. It may serve as a model to educate and improve nutrition among community-dwelling older people at risk of mobility limitations.

**Supplementary Information:**

The online version contains supplementary material available at 10.1007/s41999-020-00438-4.

## Introduction

Ageing is associated with decreased physical activity, decline in lean body mass and loss of appetite, which concur to reducing physical function and performance leading to various negative health outcomes [1, 2]. The age-related loss of physical performance often results from multiple clinical and subclinical conditions such as frailty and sarcopenia [3, 4]. Frailty is a multifactorial geriatric syndrome characterized by decreased reserve and diminished resistance to stressors [5]. Weight loss, muscle weakness, exhaustion, slow walking speed and low physical activity are symptoms and signs of phenotypic frailty [6]. Sarcopenia is closely related to frailty and is characterized by low muscle strength, low muscle mass and poor muscle quality as well as reduced physical performance [4, 7]. Both sarcopenia and frailty increase the risk of falls, mobility limitations, disability, institutionalization, and mortality [4, 6]. Malnutrition, in particular inadequate intake of protein, is a key contributor to both conditions [8, 9]. Indeed, also poor nutrition increases the risk of falls, institutionalization, use of health services and mortality, whereas good nutrition supports healthy and active aging [2, 10–12]. Nutrition is, thus, a key modifiable target to foster successful ageing.

In previous studies, short-term interventions combining nutrition with exercise have proven to be effective in improving physical function, performance and lean mass in older people [13–16].

The multi-center, Innovative Medicine Initiative (IMI)- funded project “Sarcopenia and Physical Frailty in Older People: Multicomponent Treatment Strategies” (SPRINTT) is the first long-term large-scale randomized controlled trial (RCT) focused on physical frailty and sarcopenia in community-living frail older Europeans. The primary objective of SPRINTT is to determine the effectiveness of combined physical activity and nutrition at preventing mobility disability in at-risk older people [17, 18]. Here, we describe the methods and feasibility of the nutritional intervention carried out within the SPRINTT RCT. We also illustrate how nutrition interventionists identified participants at risk of malnutrition and the lessons learnt from the nutrition intervention.

## Methods

### Overall trial design

The SPRINTT RCT was carried out in 11 European countries (Austria, Czech Republic, Finland, France, Germany, Great Britain, Iceland, Italy, the Netherlands, Poland, and Spain) with 16 study sites. The Università Cattolica del Sacro Cuore (Rome, Italy) acted as the coordinator of the study. Rationale, design and methods including power estimation of the SPRINTT RCT are detailed elsewhere [17]. Exclusion criteria are presented in supplementary Table 1. SPRINTT was approved by national ethics committees and all participants provided a written informed consent prior to enrolment. The trial is registered in ClinicalTrials.gov (NCT02582138).

**Table 1:** Questions to the nutrition interventionists

Nutrition survey
1. How did you identify a person at risk? (body weight, MNA-SF, dietary records)
2. Were body weight loss or risk of malnutrition frequent problems?
3. What methods did you use to implement the SPRINTT dietary intervention?
(a) Individual counseling
(b) Group counseling
(c) Teaching practical aspects
(d) Using brochures, guidebooks, leaflets or other educational materials
(e) Using dietary supplements or food recommendations
(f) Order meals on wheels
(g) Contact/instruct home nursing or day care centers
(h) Follow-up phone calls
4. Did you use any country-specific dietary recommendations in the nutrition intervention?
5. How often did you usually meet the participant to resolve a nutritional problem?
6. How was the motivation of the participants?
7. How did you motivate them?
8. Do you think that such dietary intervention is feasible in a frail population?
9. How was the adherence of the participants?
10. Was the dietary intervention successful in terms of dietary intake?
11. What were the main reasons for non-adherence to the dietary intervention?
12. What are the main lessons learnt from the SPRINTT dietary intervention?

*MNA-SF* mini nutritional assessment short-form

Briefly, in the SPRINTT RCT participants were randomly allocated to one of two groups: (1) a multicomponent intervention (MCI) group that received physical activity classes twice a week, a home-based exercise program, and an individualized nutritional intervention, and (2) a healthy ageing lifestyle education (HALE) group, in which participants regularly took part in various group activities and lectures about health-related topics other than nutrition and exercise. The MCI was delivered by certified exercise trainers and dieticians/nutritionists at each trial site. The trial lasted for a minimum of 2 and a maximum of 3 years depending on participants’ start date. The primary outcome of RCT was incident mobility disability, defined as an inability to walk 400 m within 15 m without a walker or assistance from another person [19]. Secondary outcomes were changes in muscle mass, physical performance, falls, nutritional status, mood, cognition, use on health services, quality of life, cost effectiveness of the study, and mortality.

### Nutrition intervention survey

A survey about the SPRINTT nutrition intervention was sent to all nutrition interventionists at the 16 trial sites. The survey covered questions about identification of participants at risk of malnutrition, methods used to carry out the intervention, and details about the intervention. The survey questions are listed in Table 1.

## Results

All of the SPRINTT nutrition interventionists responded to the survey. All responders considered the SPRINTT nutrition intervention feasible for the target population and well received by the majority of the participants.

### Identification of SPRINTT participants at nutritional risk

**Fig. 1:**
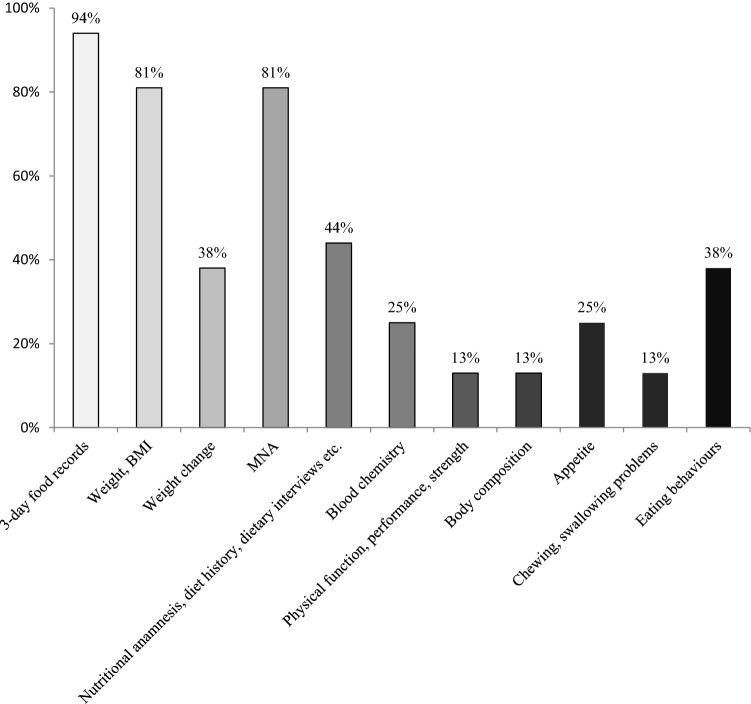
Methods used by nutrition interventionists in the SPRINTT study to identify those in nutritional risk

Participants at nutritional risk were identified by combining information from questionnaires, clinical assessments, and biomarker data presented in Table 2. Three-day food diaries, body weight or body mass index (BMI) and the Mini Nutritional Assessment (MNA) were used by more than 80% of nutrition interventionists. In addition, they used check lists and nutrition anamnesis, and carried out nutritional interviews (oral and questionnaires) about diet history, general dietary habits, possible allergies or intolerances, cooking and grocery shopping habits or food services used (meals on wheels, eating out, etc.) and other factors that might influence individual nutrition (see Fig. 1).

**Table 2:** Clinical and laboratory variables assessed in the SPRINTT trial

3-day food diaries
Mini Nutritional Assessment Short Form (MNA-SF)
Current body weight, height, and body mass index
Body composition (whole-body DXA)
Self-reported recent weight loss or weight gain
Blood analysis including albumin, cholesterol, glucose level, vitamin D, iron
Functional status
General health status
Diseases or clinical conditions affecting diet

On the basis of this information, nutrition interventionists drafted a nutritional plan in collaboration with the participants to complement and improve the participant diet as needed to meet the SPRINTT nutrition goals and to ensure good diet quality.

Body weight loss or malnutrition were named as major problems only in three study sites, whereas overweight and obesity were more common. Poor diet quality and suboptimal protein intake were commonly encountered problems.

### Nutrition intervention in the SPRINTT-study

The nutrition intervention in SPRINTT was carried out mainly through individual-tailored nutritional counselling. The participants filled in 3-day food diaries prior to meetings with a trained research nutritionist/dietician, who analyzed the diaries. The intervention had three predefined goals: a total daily energy intake of 25–30 kcal/kg body weight (BW) depending on participants’ needs, health, weight and nutritional status, (2) a daily protein intake of at least 1.0–1.2 g/ kg BW; (3) vitamin D serum levels were targeted to be at least 75 mmol/L (30 ng/mL).

The nutrition intervention was not standardized in detail, but nutrition interventionists sought to improve participants’ overall diet quality, taking into account a sufficient intake of micronutrients, good quality of fats, promotion of fruit and vegetable consumption, fiber and sufficient intake of liquids, as well as proper meal frequency. In addition, protein intake timing with daily exercise was encouraged. Nutrition interventionists also gave counseling related to special diets and other issues encountered during the trial addressing specific nutritional issues (e.g., irritable bowel syndrome, food allergies, diabetes, obesity, undernutrition, weight loss, weight gain, etc.). The general idea of nutritional counselling was not to change one’s dietary habits completely, but to complement existing diets, when, for example the energy or protein provision was low or diet quality was poor.

Other means of nutrition intervention (not obligatory) included the organization of group activities (Table 3), for example small group gatherings for discussions about food or nutrition-related themes, tasting of protein rich products, or taking a small group to a local shop to learn about good food alternatives or protein-rich products. Many sites also produced their own brochures or booklets about nutrition-related themes or used existing materials when available. Teaching practical nutrition related aspects was also a commonly used method. Nutrition interventionists kept close contact with participants via phone and followed them up, especially at times of increased risk for malnutrition due to weight loss, cognitive issues, during recovery from illness or surgery, and other nutrition-related problems.

**Table 3:** Methods used by the nutrition interventionists in SPRINTT sites to carry out the nutrition intervention

SPRINTT sites	Methods							
	Individual counseling	Group counseling	Teaching practical aspects	Brochures, leaflets, educational materials	Recommending or using dietary supplements	Contact home care or day care centers	Follow-up phone calls	Follow-up monitoring
Università Cattolica del Sacro Cuore, Rome	x		x		x			
IRCCS INRCA, Ancona	x		x		x			x
University of Parma	x		x	x	x		x	x
University Hospital Getafe	x		x		x			x
University Hospital Ramon y Cajal Madrid	x	x	x	x	x	x	x	x
CHU-Toulouse	x			x	x		x	
CHU-Limoges	x		x	x	x		x	x
Charles University, Prague	x	x	x	x	x		x	x
Silesian Hospital, Opava	x	x			x		x	
Jagiellonian University Medical College, Krakow	x	x	x	x	x		x	x
Friedrich-Alexander University, Erlangen-Nürnberg	x	x	x	x	x		x	x
Maastricht University Medical Center	x	x	x	x	x		x	x
University of Helsinki	x	x	x	x	x		x	x
Diabetes Frail, Medici Medical Practice, Luton	x	x	x	x	x		x	
Medical University of Graz	x	x		x	x	x		
Lanspitali University Hospital, Reykjavik	x	x	x	x	x		x	x
Percentage, %	16 (100%)	10 (63%)	13 (81%)	12 (75%)	16 (100%)	2 (13%)	12 (75%)	11 (69%)

Nutritional supplements were recommended for participants with low vitamin D status or suspected micronutrient deficiency. Protein drinks or supplements were also sometimes recommended to achieve the goal of SPRINTT protein intake. The participants visited nutrition interventionists 2–12 times a year, more often if extra support with nutrition was needed.

Nutrition interventionists followed mainly local dietary recommendations or Mediterranean diet (Italy, Spain) to ensure good diet quality and health-promoting diet (Table 4).

**Table 4:** Nutrition recommendations used in addition to the SPRINTT protocol instructions to improve overall diet quality

Research sites	Nutrition Recommendation
Graz, Austria	Austrian
Getafe, Spain	Australian
Madrid, Spain	Mediterranean
Rome, Italy	Mediterranean
Parma, Italy	Italian guidelines, Mediterranean
Nürnberg, Germany	German Nutrition Society recommendations
Krakow, Poland	Polish
Luton, UK	UK recommendations
Helsinki, Finland	Finnish (Nordic) recommendations
Reykjavik, Iceland	Icelandic recommendations
Prague, Czech	DACH (German-Austria-Swizerland) Recommendations
Opava, Czech	DACH (German-Austria-Swizerland) Recommendations
Limogen, France	French
Toulouse, France	French & nutrition support for protein-energy malnutrition, Nutrition guide for + 55 y
Maastricht, NL	Netherlands Nutrition Centre
Ancona, Italy	-

### Feasibility of nutrition intervention

The nutrition intervention in SPRINTT allowed flexibility in delivering the nutrition intervention and messages to participants. According to responding nutritionists, intervention was feasible, important and well received by the majority of the participants. Most of them were able to improve their nutritional habits and diet quality, and to increase protein intake.

However, all SPRINTT sites had also participants who did not regularly take part in the nutrition intervention. Reasons for non-adherence included lack of interest or considering nutrition counselling useless, not liking guidelines, depression, lack of willpower, old habits, cognitive issues, or problems with hearing or eyesight. In some cases, a large family cooking together was seen as an obstacle to change one’s dietary habits.

### Lessons learnt of SPRINTT nutrition intervention

According to responders, participants’ motivation was the most important prerequisite for the successful delivery of the SPRINTT nutritional intervention. Modifying established habits in older people can be challenging. Therefore, a major aspect of the intervention was to motivate participants to fully participate in nutrition sessions and to monitor whether the recommended dietary changes were implemented.

Many of the nutrition interventionists mentioned that intervention had to be individualized and practical, started before malnutrition occurs, and potential memory problems taken into account. Moreover, giving participants written feedback and educational materials of main aspects of counselling sessions was seen of outmost importance by many responders.

Besides memory problems, also other specific characteristics of this population were important to consider: loneliness and insufficient economic means may be substantial underlying obstacles for following nutrition advice. Encouraging the participants and giving positive feedback was considered very important as well as reporting benefits to the participants when even small progress was observed. Responders also mentioned that respecting cultural or specific diets of the participants was considered essential.

## Discussion

The SPRINTT RCT is a model for delivering nutritional intervention to a heterogeneous group of frail community-dwelling older people with functional limitations. It was well received by the majority of the participants and feasible for the target group. The nutrition intervention was mainly carried out using tailored nutrition counselling, but also other means of delivering the intervention were successfully used. Compared with a standard nutrition prescription, an individualized protocol to diagnose malnutrition and follow-up by tailored nutrition counselling helped achieve nutritional targets more effectively in spite of diversity of population in nutritional habits and in some cases reluctance to accept changes [20].

Previous RCTs aimed at improving functional status, performance, muscle strength or mass of older people with exercise and nutrition have mostly used protein supplementation as nutritional strategy [14]. In a systematic review and meta-analysis of 17 RCTs, Liao et al. [4] reported that older participants had substantially greater gains of lean mass and leg strength when protein supplementation and resistance exercise training were combined compared with resistant exercise alone. Results from more recent similar studies also support these findings [15, 16, 21, 22]. Although aiming at increasing protein intake, SPRINTT was based on a different approach: providing long-term nutrition counselling to the participants allowed them to learn and change their dietary habits over a longer period of time (2–3 years). This approach holds the potential of promoting a longer lasting behavioral change as compared with merely using protein supplements—the benefits of which are often lost after the supplementation ends. The role of trained nutrition professionals is suggested to be essential in both primary and secondary prevention to decrease the risk of non-communicable diseases, including sarcopenia and malnutrition [23]. Indeed, the importance of nutrition therapy for vulnerable groups and the added value of follow-up by a nutrition professional and educational approach have recently been demonstrated [24, 25]. The SPRINTT nutritional strategy—based on individualized counselling and proposing foods familiar to the participants could in the long run be more feasible than dietary supplements for community-dwelling older adults.

Nutrition interventionists emphasized individualized and practical advice. Future studies should include hands-on nutrition such as self-cooking, guided shopping tours to identify healthy foods, and teaching participants to consume easy and fast protein-rich snacks along with other practical activities. Although individual counselling was considered feasible and important, small group activities were also commonly used and their feasibility for delivering nutrition interventions should be explored in future intervention studies. Some nutrition interventionists suggested that dietary intervention should be started as early as possible, because existing dietary habits may be difficult to change. Prevention of frailty and sarcopenia through nutrition and exercise in “young-old”people (60–75 years) may be an important and interesting research area to be considered in the future.

There are several strengths and weaknesses in the SPRINTT nutrition intervention. The main strength of this study is that it was feasible for the large and heterogeneous target population. An individualized nutritional intervention program was based on the nutritional needs identified at baseline using various methods. The intervention was tailored according to individual’s needs, preferences, culture, and physical conditions. The intervention was focused on achieving adequate energy and protein intake and diet quality, thereby reducing the progression of sarcopenia and frailty. With the heterogeneity and varying nutrition-related problems of the population this was probably the most feasible way to carry out the trial. However, the non-standardized nutrition intervention may also be seen as a weakness; intervention may not have been equally intensive at all study sites; in some sites participants received much more frequent and intensive counselling and other nutrition-related activities compared to other sites. This could affect the overall success of the trial. However, this will also tell about real-life effectiveness.

Dietary records are subject to under- or over-reporting, in particular if a participant has cognitive problems. Although in SPRINTT, participants with cognitive impairment (Mini Mental State Examination < 24/30) at baseline were excluded, cognitive status may have declined during the trial. However, we tried to overcome these problems by asking assistance of spouses as necessary for completing the food records. Moreover, nutrition interventionists gave the participants both oral and written instructions how to fill in the food diaries and upon return of the food records asked them to check amounts and types of foods eaten. Food diaries are considered one of the best ways to evaluate dietary intakes in older people, because they do not rely on short-term memory [26]. Therefore, although food diaries may not have been completely accurate, they still gave a reasonably good estimate of the diet.

In conclusion, the SPRINTT nutrition intervention was feasible and allowed flexibility to the varying needs and cultural differences of this heterogeneous population of frail, older Europeans. It may serve as a model to educate and improve nutrition among community-dwelling older people at risk of mobility limitations.

## Supplementary Information

Below is the link to the electronic supplementary material.

Supplementary file1 (DOCX 36 KB)
