# Supplementary material for: The sarcopenia and physical frailty in older people: multi-component treatment strategies (SPRINTT) project: description and feasibility of a nutrition intervention in community-dwelling older Europeans
Source: Eur Geriatr Med. 2021 Feb 13;12(2):303–12. doi: 10.1007/s41999-020-00438-4 (PMC7990826; doi:10.1007/s41999-020-00438-4)
Supplement: Supplementary file 1 — Supplementary file1 (DOCX 36 KB) [file 41999_2020_438_MOESM1_ESM.docx]

Supplementary table 1. Exclusion criteria for the SPRNTT trial

| Permanent exclusion criteria | Inability or unwillingness to provide informed consent or accept randomization in either study group  Plans to relocate out of the study area within the next 2 years or plans to be out of the study area for more than 6 consecutive weeks in the next year  Residence in long-term care  Household member enrolled in the study  Current diagnosis of schizophrenia, other psychotic or bipolar disorder  Consumption of more than 14 alcoholic drinks per week  Difficulty communicating with the study personnel due to speech, language, or (non-corrected) hearing problems  Cognitive impairment (i.e., MMSE score <24/30)  Severe arthritis (e.g., awaiting joint replacement) that would interfere with the ability to participate fully in either study arm  Cancer requiring treatment in the past 3 years, except for non-melanoma skin cancers or cancers that have an excellent prognosis (e.g., the early stage breast or prostate cancer)  Lung disease requiring regular use of supplemental oxygen  Inflammatory conditions requiring regular use of oral or parenteral corticosteroid agents  Severe cardiovascular disease [including New York Heart Association (NYHA) class III or IV congestive heart failure, clinically significant valvular disease, history of cardiac arrest, presence of an implantable defibrillator, or uncontrolled angina]  Peripheral arterial disease Lériche–Fontaine stage 3 or 4  Upper and/or lower extremity amputation  Parkinson’s disease or other progressive neurological disorder  Renal disease requiring dialysis  Chest pain, severe shortness of breath, or occurrence of other safety concerns during baseline 400-m walk test  Current participation in a structured physical activity program  Current enrolment in another clinical trial involving lifestyle, nutrition, or pharmaceutical interventions  Other medical, psychiatric, or behavioral factors that in the judgment of the investigator may interfere with the study participation or the ability to autonomously follow either the MCI or the HALE programs  Other illness of such severity that life expectancy is expected to be less than 12 months  Clinical judgment concerning safety or non-compliance |
| --- | --- |
| Temporal exclusion criteria | Uncontrolled hypertension (systolic blood pressure >200 mmHg, or diastolic blood pressure >110 mmHg)  Uncontrolled diabetes with recent weight loss, diabetic coma, or frequent hypoglycemia  Hip fracture, hip or knee replacement, or spinal surgery in the past 6 months  Serious cardiac conduction disorder (e.g., third-degree heart block), uncontrolled arrhythmia, new Q waves within the past 6 months, or ST segment depression (> 3 mm) on the ECG  Myocardial infarction, major heart surgery (i.e., valve replacement or coronary bypass graft), stroke, deep vein thrombosis, or pulmonary embolism in the past 6 months  Use of growth hormone, estrogens, progesterone, or testosterone supplementation in the past 3 months  Current participation in physical therapy or cardiopulmonary rehabilitation |
